# Supplementary material for: Nilotinib, an approved leukemia drug, inhibits smoothened signaling in Hedgehog-dependent medulloblastoma
Source: PLoS One. 2019 Sep 20;14(9):e0214901. doi: 10.1371/journal.pone.0214901 (PMC6754133; doi:10.1371/journal.pone.0214901)
Supplement: S1 Fig — (DOCX) [file pone.0214901.s001.docx]

**
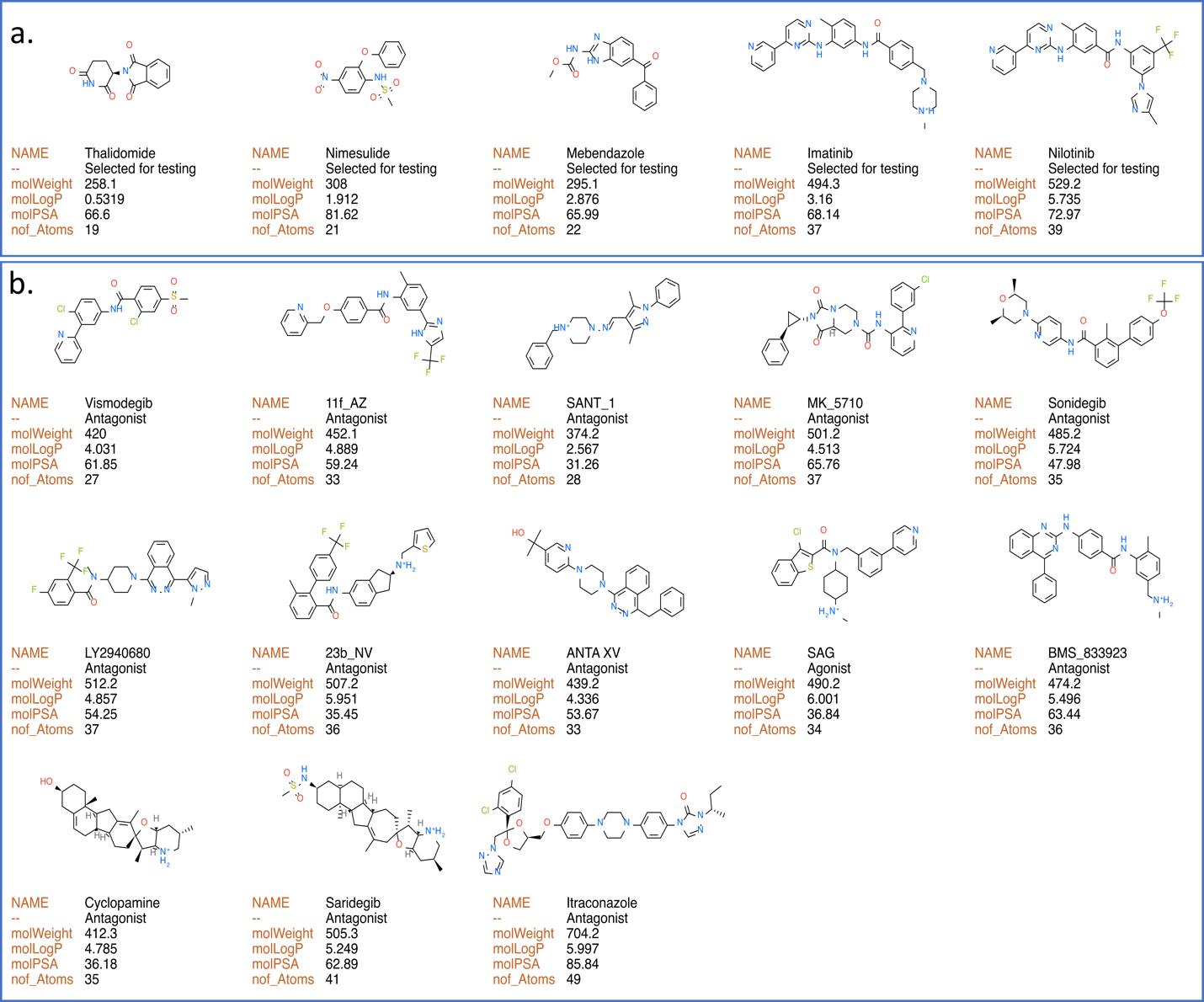
**

**S1 Figure: Calculated properties of SMO Modulators and Drugs Selected for Experimental Validation. (a)** Drugs selected for *in-vitro* validation **(b)** SMO modulators used in *in-silico* validation
