# Supplementary material for: Nilotinib, an approved leukemia drug, inhibits smoothened signaling in Hedgehog-dependent medulloblastoma
Source: PLoS One. 2019 Sep 20;14(9):e0214901. doi: 10.1371/journal.pone.0214901 (PMC6754133; doi:10.1371/journal.pone.0214901)
Supplement: S2 Fig — (DOCX) [file pone.0214901.s002.docx]

**
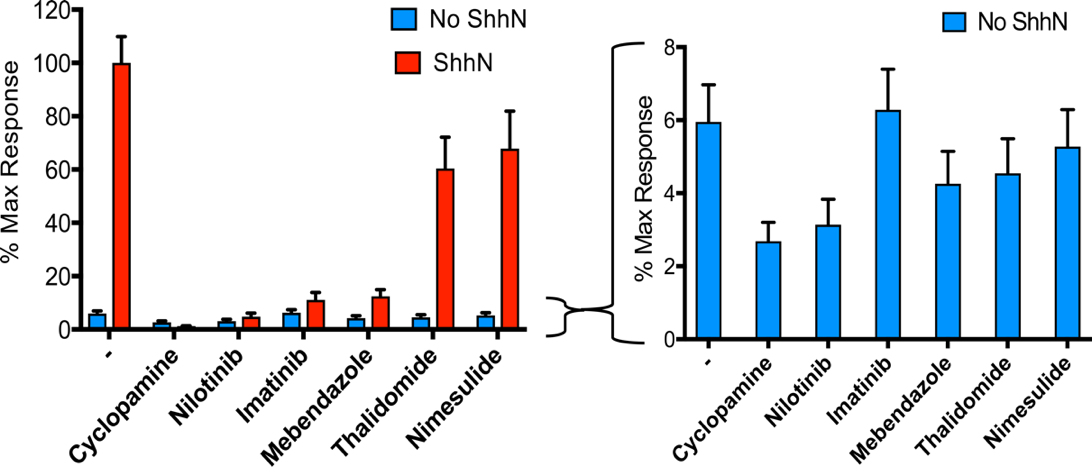
**

**S2 Figure:** **Effect of test drugs on Hh pathway activity with and without ShhN**. The inhibition of Hh pathway activity in NIH3T3 Gli-RE cells after exposure of drugs (10 µM) for 24 hours with and without ShhN-conditioned media confirms the selective effect of drugs on the pathway and Gli activity. (Mean ± SD)
