# Supplementary material for: Nilotinib, an approved leukemia drug, inhibits smoothened signaling in Hedgehog-dependent medulloblastoma
Source: PLoS One. 2019 Sep 20;14(9):e0214901. doi: 10.1371/journal.pone.0214901 (PMC6754133; doi:10.1371/journal.pone.0214901)
Supplement: S3 Fig — (DOCX) [file pone.0214901.s003.docx]

**
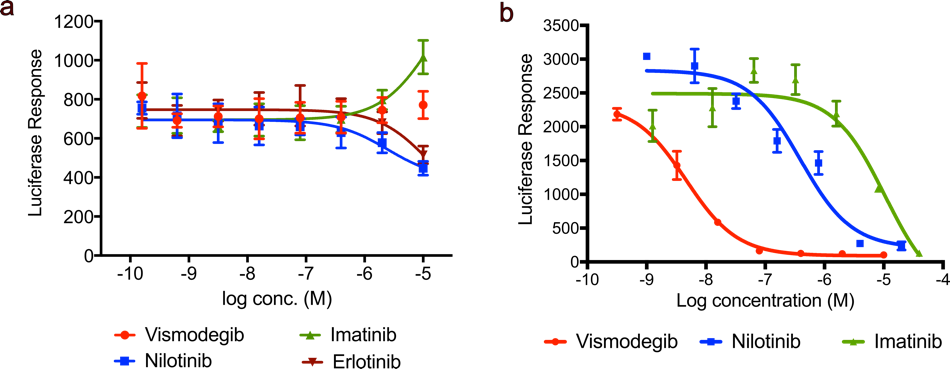
**

**S3 Figure: Nilotinib, Imatinib, and Vismodegib inhibit luciferase reporter activity in an Hh-dependent manner. (a)** The drugs were tested at different concentrations in HEK293s cells stably transfected with firefly luciferase under control of cAMP response element (CRE) (pGL4.29[luc2P/CRE/Hygro], Promega). The lack of dose-dependent effect is observed. **(b)** By contrast, the drugs demonstrate robust and dose-dependent inhibition of luminescence in NIH3T3 Gli-RE cells transfected with firefly luciferase under the transcriptional control of Gli response element (8x GliRE-luc). Raw, non-normalized luminescence values are presented, to facilitate the assessment of the extent of inhibition. Shown is a single representative experiment out of the multiple experiments performed on different days. (Mean ± SD)
