# Supplementary material for: Nilotinib, an approved leukemia drug, inhibits smoothened signaling in Hedgehog-dependent medulloblastoma
Source: PLoS One. 2019 Sep 20;14(9):e0214901. doi: 10.1371/journal.pone.0214901 (PMC6754133; doi:10.1371/journal.pone.0214901)
Supplement: S4 Fig — (DOCX) [file pone.0214901.s004.docx]

**
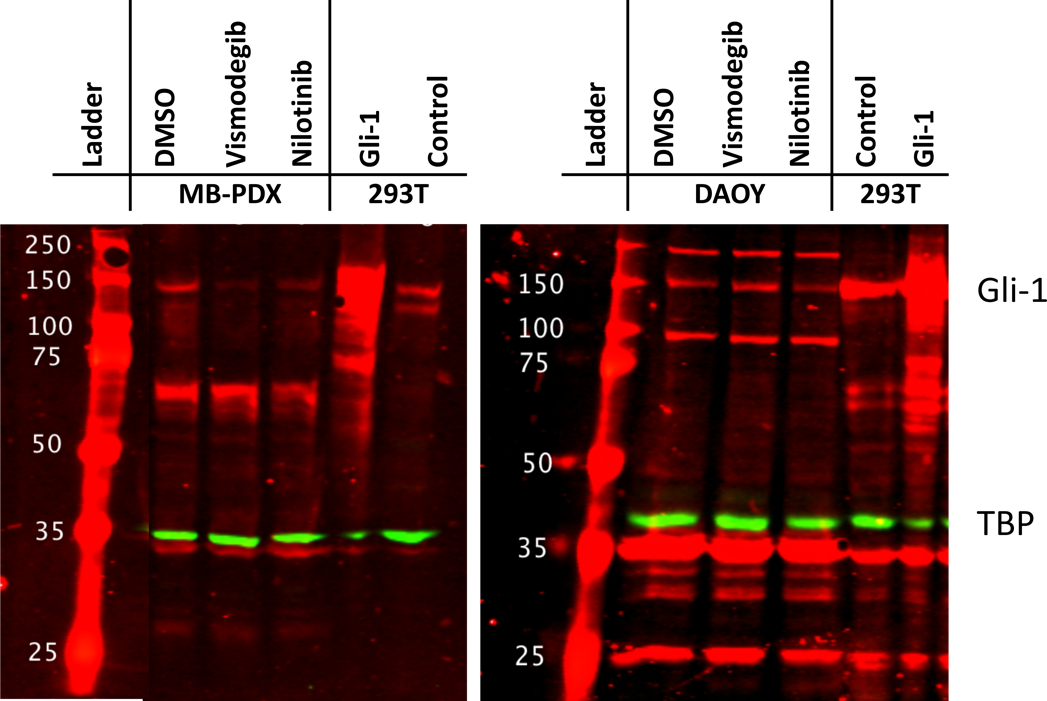
**

**S4 Figure:** Original Western Blot images presented as Figure 6(f) in the manuscript for quantitation of Gli-1 (MW – 150) after treatment with DMSO or 5 μM Nilotinib/Vismodegib for 24 hours in MB-PDX and DAOY cells using Odyssey IR imaging system. Gli-1 – red; Tata Binding Protein (TBP, loading control) – Green
