# Supplementary material for: Nilotinib, an approved leukemia drug, inhibits smoothened signaling in Hedgehog-dependent medulloblastoma
Source: PLoS One. 2019 Sep 20;14(9):e0214901. doi: 10.1371/journal.pone.0214901 (PMC6754133; doi:10.1371/journal.pone.0214901)
Supplement: S5 Fig — (DOCX) [file pone.0214901.s005.docx]

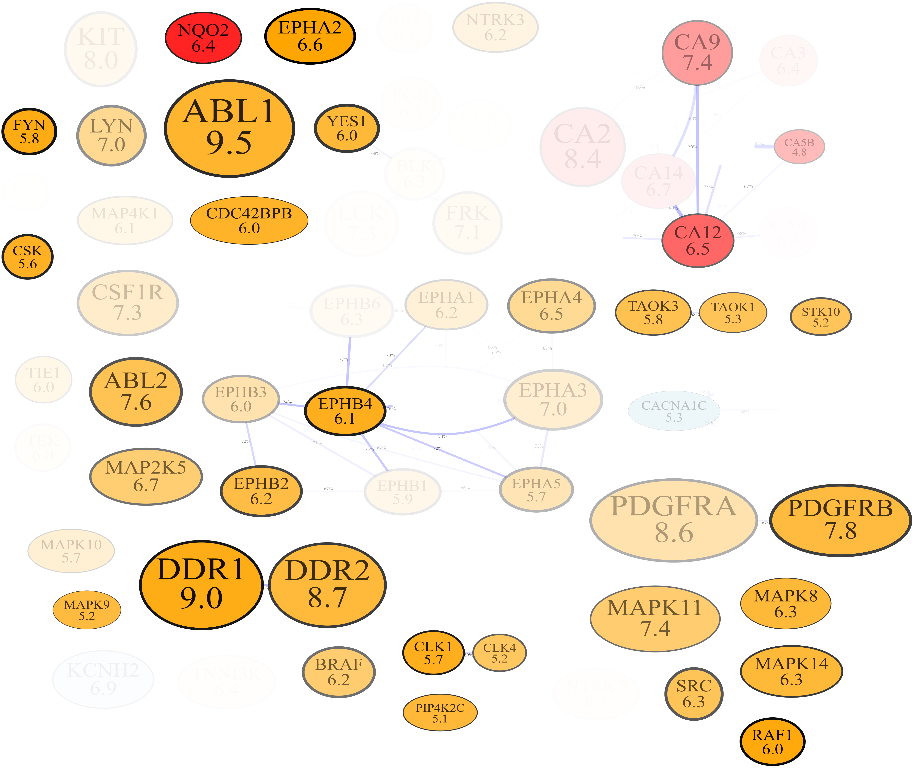


**S5 Figure: CNS-expression-weighted map of all Nilotinib targets[1]**. Experimental target(s) of nilotinib, values under the target gene names are the pAct (-Log(IC50/Ki/Kd)) of nilotinib to the targets. The opacity of each target corresponds to its median expression level (RPKM) in central nervous system.

**Reference:**

1. Shi D, Khan F, Abagyan R. Extended Multitarget Pharmacology of Anticancer Drugs. J Chem Inf Model. 2019 Jun 24;59(6):3006–17.
