# Supplementary material for: Nilotinib, an approved leukemia drug, inhibits smoothened signaling in Hedgehog-dependent medulloblastoma
Source: PLoS One. 2019 Sep 20;14(9):e0214901. doi: 10.1371/journal.pone.0214901 (PMC6754133; doi:10.1371/journal.pone.0214901)
Supplement: S6 Fig — (DOCX) [file pone.0214901.s006.docx]

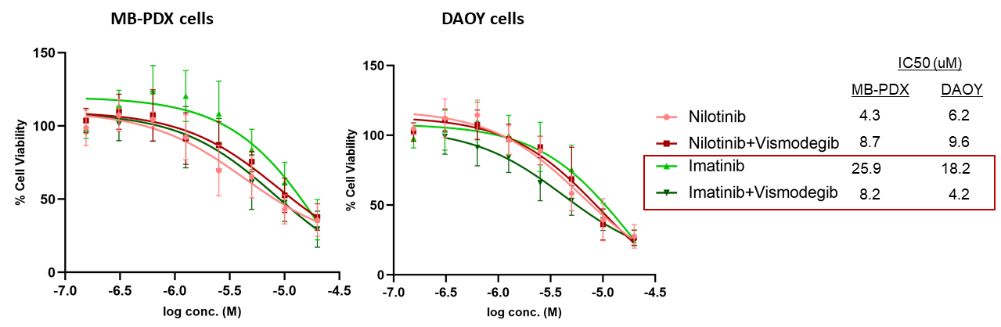


**S6 Figure: Cell viability in Medulloblastoma Cells (MB-PDX and DAOY) [n=3].** MB-PDX and DAOY cells were treated with different concentrations of Nilotinib and Imatinib with and without Vismodegib (0.5 uM). The cell viability was measured after 72 hours of treatment using alamar blue. (Mean ± SD)
