# Supplementary material for: Nilotinib, an approved leukemia drug, inhibits smoothened signaling in Hedgehog-dependent medulloblastoma
Source: PLoS One. 2019 Sep 20;14(9):e0214901. doi: 10.1371/journal.pone.0214901 (PMC6754133; doi:10.1371/journal.pone.0214901)
Supplement: S7 Fig — (DOCX) [file pone.0214901.s007.docx]

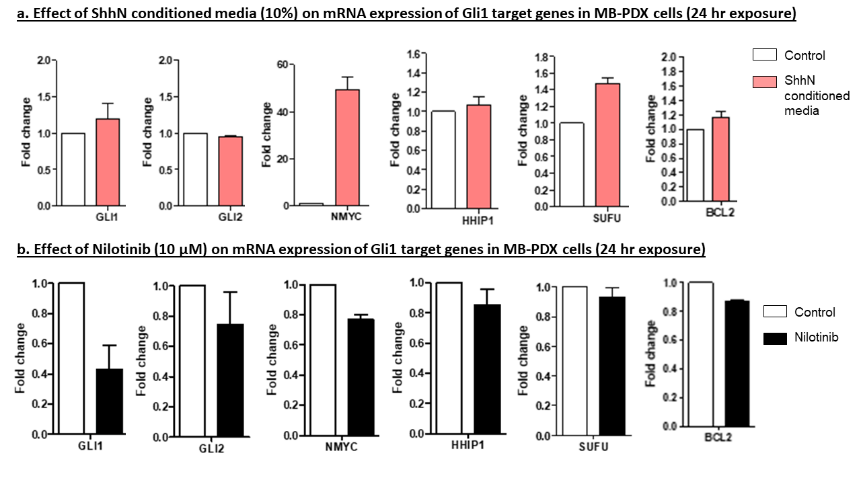


**S7 Figure:** **Effect of ShhN (Hh pathway activator) and Nilotinib (SMO antagonist) on mRNA expression of Gli target genes in MB-PDX cells (*in-vitro*).** Each panel shows two bars corresponding to control (vehicle) and treatment for expression levels of target gene (gene name specified under each panel). Y-axes show the relative expression levels of mRNA of target genes with respect to mRNA of GAPDH gene.
