# Supplementary material for: Nilotinib, an approved leukemia drug, inhibits smoothened signaling in Hedgehog-dependent medulloblastoma
Source: PLoS One. 2019 Sep 20;14(9):e0214901. doi: 10.1371/journal.pone.0214901 (PMC6754133; doi:10.1371/journal.pone.0214901)
Supplement: S1 Table — (PDF) [file pone.0214901.s008.pdf]

Table S1 - List of Top Scoring Molecules in SMO Docking			
	**Chemical structure**	**Name**	**Docking score**
1	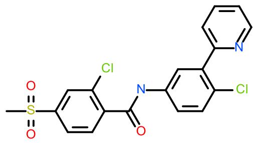	Vismodegib	-43.40
2	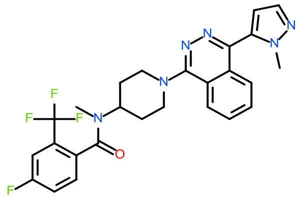	LY2940680	-43.28
3	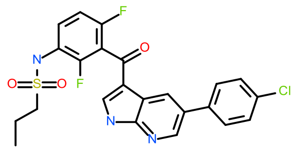	Vemurafenib	-41.20
4	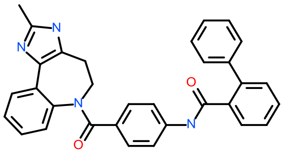	Conivaptan	-40.61
5	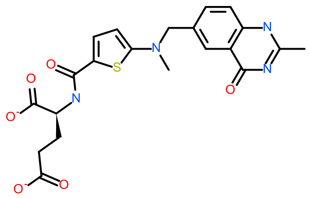	Raltitrexed	-40.58
6	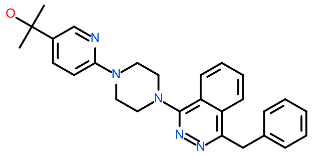	Anta_XV	-39.90
7	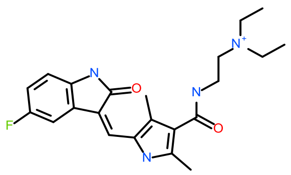	Sunitinib	-38.17

s

8	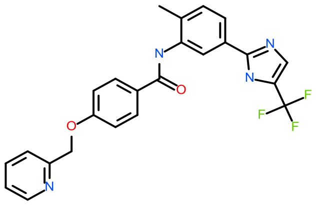	AZ_p38	-37.66
9	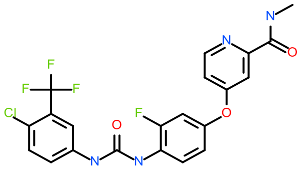	Regorafenib	-37.49
10	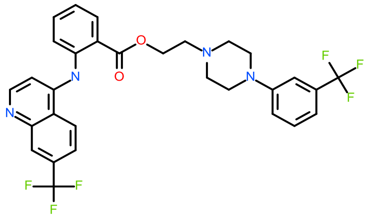	Antrafenine	-37.43
11	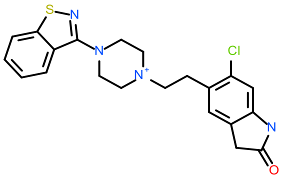	Ziprasidone	-37.13
12	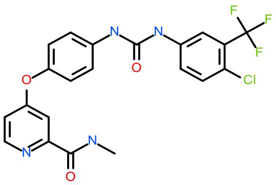	Sorafenib	-37.06
13	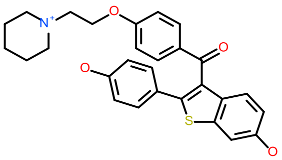	Raloxifene	-36.71
14	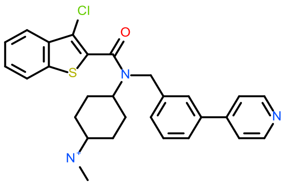	SAG	-36.50

15	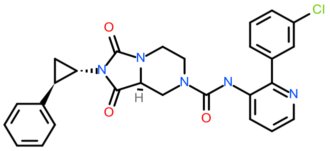	MK_5710	-36.09
16	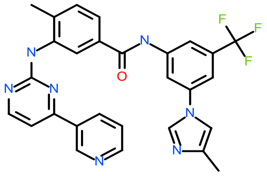	Nilotinib	-35.98
17	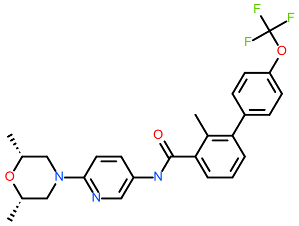	Sonidegib	-35.65
18	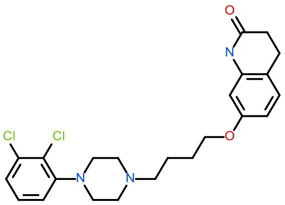	Aripiprazole	-34.84
19	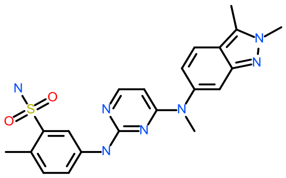	Pazopanib	-34.84
20	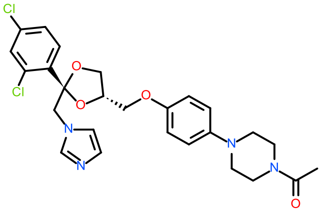	Ketoconazole	-34.71
21	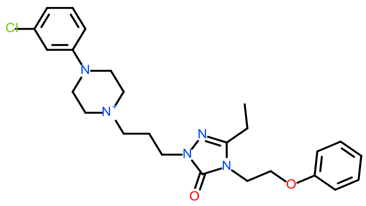	Nefazodone	-34.66

22	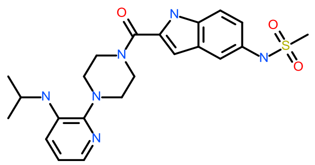	Delavirdine	-34.53
23	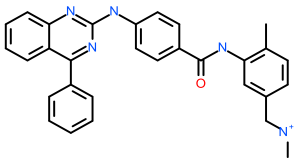	BMS_833923	-34.44
24	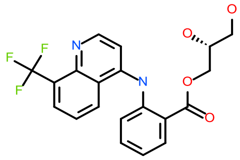	Floctafenine	-34.43
25	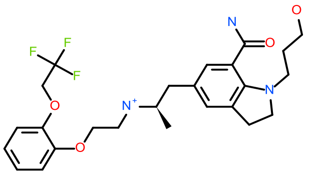	Silodosin	-34.07
26	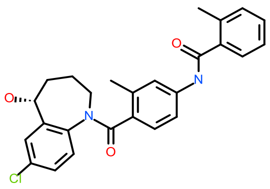	Tolvaptan	-33.97
27	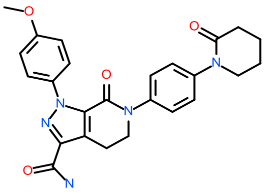	Apixaban	-33.93
28	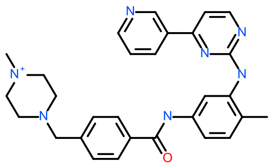	Imatinib	-33.88

29	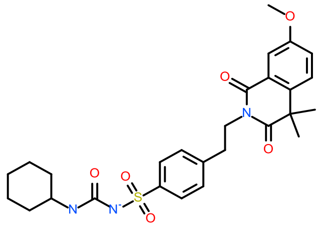	Gliquidone	-33.85
30	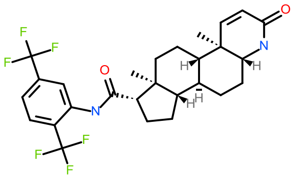	Dutasteride	-33.76
31	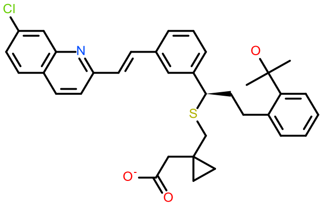	Montelukast	-33.73
32	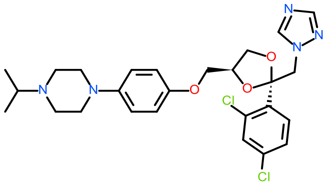	Terconazole	-33.59
33	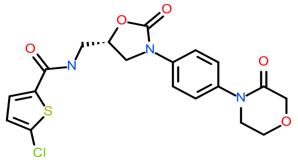	Rivaroxaban	-33.42
34	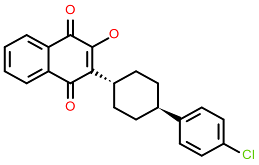	Atovaquone	-33.27
35	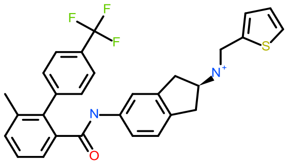	Novartis_21b	-32.98

36	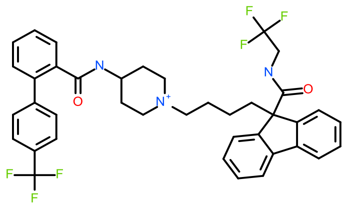	Lomitapide	-32.96
37	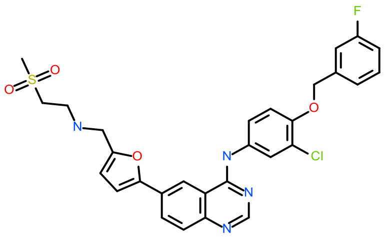	Lapatinib	-32.87
38	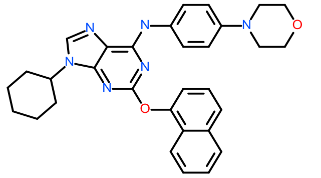	Purmorphamine	-32.47
39	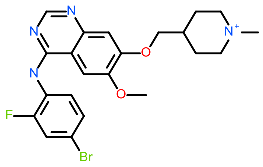	Vandetanib	-32.32
40	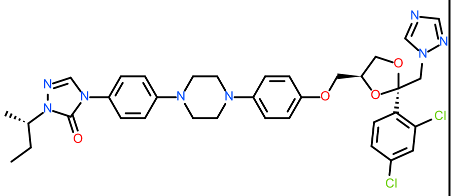	Itraconazole	-32.30
41	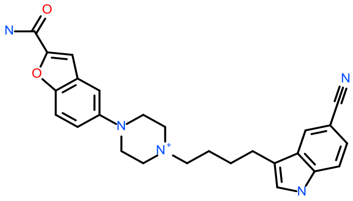	Vilazodone	-32.29
42	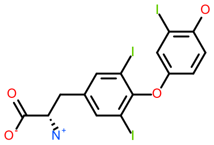	Liothyronine	-32.21

43	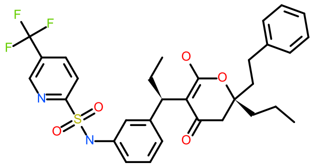	Tipranavir	-32.19
44	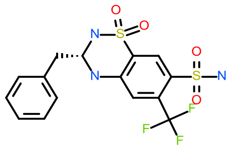	Bendroflumethiazid	-32.15
45	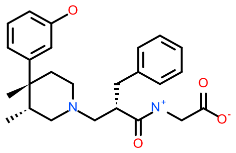	Alvimopan	-32.13
46	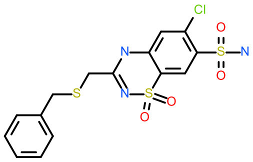	Benzthiazide	-32.06
47	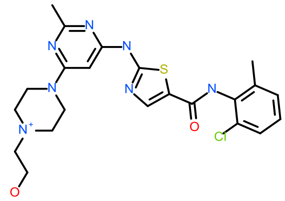	Dasatinib	-31.98
48	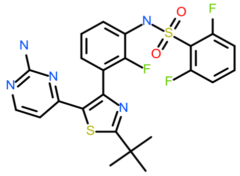	Dabrafenib	-31.82
49	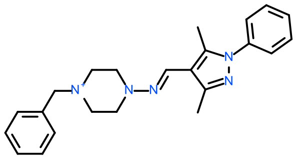	Sant_1	-31.76

50	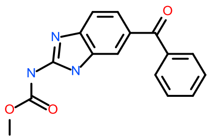	Mebendazole	-28.56
