# Supplementary material for: Nilotinib, an approved leukemia drug, inhibits smoothened signaling in Hedgehog-dependent medulloblastoma
Source: PLoS One. 2019 Sep 20;14(9):e0214901. doi: 10.1371/journal.pone.0214901 (PMC6754133; doi:10.1371/journal.pone.0214901)
Supplement: S2 Table — (DOCX) [file pone.0214901.s009.docx]

**S2 Table - List of Imatinib Targets and the K_d_/K_i_ values**

|  | **Target** | **uniprot ID** | **Protein Type** | **Value (nM)** | **Reference** |
| --- | --- | --- | --- | --- | --- |
| 1 | Epithelial discoidin domain-containing receptor 1 | DDR1_HUMAN | RTK | 0.7 | [1] |
| 2 | Tyrosine-protein kinase ABL1 | ABL1_HUMAN | nRTK | 1.1 | [1] |
| 3 | Abelson tyrosine-protein kinase 2 | ABL2_HUMAN | nRTK | 10 | [1] |
| 4 | Macrophage colony-stimulating factor 1 receptor | CSF1R_HUMAN | RTK | 11 | [1] |
| 5 | Mast/stem cell growth factor receptor Kit | KIT_HUMAN | RTK | 13 | [1] |
| 6 | Platelet-derived growth factor receptor beta | PGFRB_HUMAN | RTK | 14 | [1] |
| 7 | Discoidin domain-containing receptor 2 | DDR2_HUMAN | RTK | 15 | [1] |
| 8 | Carbonic anhydrase 2 | CAH2_HUMAN | Enzyme | 30.2 | [2] |
| 9 | Platelet-derived growth factor receptor alpha | PGFRA_HUMAN | RTK | 31 | [1] |
| 10 | Carbonic anhydrase 1 | CAH1_HUMAN | Metalloenzyme | 31.9 | [2] |
| 11 | Tyrosine-protein kinase Lck | LCK_HUMAN | nRTK | 40 | [1] |
| 12 | Carbonic anhydrase 9 | CAH9_HUMAN | Metalloenzyme | 75.7 | [2] |

**References:**

1. Davis MI, Hunt JP, Herrgard S, Ciceri P, Wodicka LM, Pallares G, et al. Comprehensive analysis of kinase inhibitor selectivity. Nat Biotechnol. 2011;29(11):1046–1051.

2. Parkkila S, Innocenti A, Kallio H, Hilvo M, Scozzafava A, Supuran CT. The protein tyrosine kinase inhibitors imatinib and nilotinib strongly inhibit several mammalian α-carbonic anhydrase isoforms. Bioorg Med Chem Lett. 2009 Aug;19(15):4102–6.
