# Supplementary material for: Nilotinib, an approved leukemia drug, inhibits smoothened signaling in Hedgehog-dependent medulloblastoma
Source: PLoS One. 2019 Sep 20;14(9):e0214901. doi: 10.1371/journal.pone.0214901 (PMC6754133; doi:10.1371/journal.pone.0214901)
Supplement: S3 Table — (DOCX) [file pone.0214901.s010.docx]

**S3 Table - List of Nilotinib Targets and the K_d_/K_i_ values**

|  | **Target** | **Uniprot ID** | **Protein Type** | **Value (nM)** | **Reference** |
| --- | --- | --- | --- | --- | --- |
| 1 | Epithelial discoidin domain-containing receptor 1 | DDR1_HUMAN | RTK | 1.1 | [1] |
| 2 | Carbonic anhydrase 2 | CAH2_HUMAN | Metalloenzyme | 4.1 | [2] |
| 3 | Mitogen-activated protein kinase MLT | MLTK_HUMAN | nRTK | 11 | [1] |
| 4 | Tyrosine-protein kinase ABL1 | ABL1_HUMAN | nRTK | 13 | [1] |
| 5 | Abelson tyrosine-protein kinase 2 | ABL2_HUMAN | nRTK | 26 | [1] |
| 6 | Mast/stem cell growth factor receptor Kit | KIT_HUMAN | RTK | 29 | [1] |
| 7 | Carbonic anhydrase 1 | CAH1_HUMAN | Metalloenzyme | 29.3 | [2] |
| 8 | Discoidin domain-containing receptor 2 | DDR2_HUMAN | RTK | 33 | [1] |
| 9 | Mitogen-activated protein kinase 11 | MK11_HUMAN | nRTK | 36 | [1] |
| 10 | Ephrin type-A receptor 8 | EPHA8_HUMAN | RTK | 37 | [1] |
| 11 | Carbonic anhydrase 9 | CAH9_HUMAN | Metalloenzyme | 41.9 | [2] |
| 12 | Macrophage colony-stimulating factor 1 receptor | CSF1R_HUMAN | RTK | 45 | [1] |
| 13 | Tyrosine-protein kinase Lck | LCK_HUMAN | nRTK | 47 | [1] |
| 14 | Platelet-derived growth factor receptor beta | PGFRB_HUMAN | RTK | 73 | [1] |
| 15 | Tyrosine-protein kinase FRK | FRK_HUMAN | nRTK | 86 | [1] |
| 16 | Carbonic anhydrase 7 | CAH7_HUMAN | Metalloenzyme | 99 | [2] |
| 17 | Tyrosine-protein kinase Lyn | LYN_HUMAN | nRTK | 100 | [1] |
| 18 | Platelet-derived growth factor receptor alpha | PGFRA_HUMAN | RTK | 180 | [1] |

**References:**

1. Davis MI, Hunt JP, Herrgard S, Ciceri P, Wodicka LM, Pallares G, et al. Comprehensive analysis of kinase inhibitor selectivity. Nat Biotechnol. 2011;29(11):1046–1051.

2. Parkkila S, Innocenti A, Kallio H, Hilvo M, Scozzafava A, Supuran CT. The protein tyrosine kinase inhibitors imatinib and nilotinib strongly inhibit several mammalian α-carbonic anhydrase isoforms. Bioorg Med Chem Lett. 2009 Aug;19(15):4102–6.
