# Supplementary material for: Nilotinib, an approved leukemia drug, inhibits smoothened signaling in Hedgehog-dependent medulloblastoma
Source: PLoS One. 2019 Sep 20;14(9):e0214901. doi: 10.1371/journal.pone.0214901 (PMC6754133; doi:10.1371/journal.pone.0214901)
Supplement: S4 Table — (DOCX) [file pone.0214901.s011.docx]

**S4 Table - Transcript Levels of Hh Pathway components and Nilotinib Targets in DAOY Cells (RPKM values)[1]**

| **Symbol** | | **Length** | **Unique Reads** |  | **Coverage** | **RPKM** | **Category** | |
| --- | --- | --- | --- | --- | --- | --- | --- | --- |
| SMO | | 3772 | 17089 |  | 99.73% | 31.5 | Hh-Pathway Component and Nilotinib Target | |
| PTCH1 | | 8065 | 3280 |  | 98.02% | 3.4 | Hh-Pathway Component | |
| GLI1 | | 3618 | 323 |  | 99.75% | 0.8 | Hh-Pathway Component | |
| GLI2 | | 6780 | 3525 |  | 99.37% | 3.6 | Hh-Pathway Component | |
| GLI3 | | 8228 | 17120 |  | 99.78% | 17.0 | Hh-Pathway Component | |
| SUFU | | 4994 | 3969 |  | 98.82% | 5.5 | Hh-Pathway Component | |
| ABL1 | | 5881 | 32576 |  | 99.88% | 43.9 | Nilotinib Target | |
| ABL2 | | 12244 | 11100 |  | 99.64% | 7.0 | Nilotinib Target | |
| PDGFC | | 3079 | 15777 |  | 99.51% | 47.8 | Agonist of Nilotinib Target PDGFRA | |
| CSK | | 2755 | 19629 |  | 99.49% | 42.0 | Nilotinib Target | |
| EPHB2 | | 4869 | 36403 |  | 98.34% | 53.8 | Nilotinib Target | |
| EPHB4 | | 4369 | 15812 |  | 99.50% | 23.8 | Nilotinib Target | |
| EPHA2 | | 3970 | 12390 |  | 99.04% | 17.2 | Nilotinib Target | |
| CDC42BPB | | 6701 | 29434 |  | 98.18% | 35.1 | Nilotinib Target | |
| DDR1 | | 3877 | 9118 |  | 99.61% | 17.7 | Nilotinib Target | |
| DDR2 | | 3252 | 2245 |  | 99.05% | 13.5 | Nilotinib Target | |
| MAPK14 | | 4353 | 10022 |  | 96.78% | 17.5 | Nilotinib Target | |
| MAPK9 | | 4341 | 4974 |  | 97.37% | 8.7 | Nilotinib Target | |
| MAPK8 | | 1417 | 1106 |  | 97.74% | 7.9 | Nilotinib Target | |
| MAPK11 | | 2420 | 713 |  | 98.88% | 2.1 | Nilotinib Target | |
| CSF1 | | 4249 | 3528 |  | 97.67% | 6.8 | Nilotinib Target | |
| LYN | | 4158 | 2245 |  | 95.43% | 4.2 | Nilotinib Target | |
| PDGFRB | | 5718 | 2739 |  | 99.90% | 3.5 | Nilotinib Target | |
| PDGFRA | | 6574 | 442 |  | 94.93% | 0.6 | Nilotinib Target | |
| PDGFA | | 2809 | 1446 |  | 85.12% | 3.4 | Nilotinib Target | |
| CLK1 | | 3333 | 2659 |  | 96.67% | 7.2 | Nilotinib Target | |
| FYN | | 3628 | 9402 |  | 97.41% | 20.9 | Nilotinib Target | |
| NQO2 | | 1139 | 1608 |  | 99.56% | 8.3 | Nilotinib Target | |
| TAOK1 | | 12096 | 14658 |  | 99.89% | 12.7 | Nilotinib Target | |
| TAOK2 | | 4960 | 8333 |  | 99.82% | 11.6 | Nilotinib Target | |
| TAOK3 | | 4399 | 4000 |  | 98.30% | 6.6 | Nilotinib Target | |
| YES1 | | 4685 | 3257 |  | 96.54% | 6.7 | Nilotinib Target | |
| STK10 | | 6039 | 10477 |  | 95.88% | 12.2 | Nilotinib Target | |
| CA12 | | 3992 | 1074 |  | 99.50% | 1.7 | Nilotinib Target | |
| MAP2K5 | | 2385 | 4980 |  | 97.53% | 14.2 | Nilotinib Target | |
|  | |  |  |  |  |  |  | |
|  | RPKM = reads per kilo base per million mapped reads, a representation of absolute abundance of transcripts and a measure of gene expression | | | | | | |  |

**References:**

1. Higdon R, Kala J, Wilkins D, Yan J, Sethi M, Lin L, et al. Integrated Proteomic and Transcriptomic-Based Approaches to Identifying Signature Biomarkers and Pathways for Elucidation of Daoy and UW228 Subtypes. Proteomes. 2017 Feb 3;5(4):5.
